# Nutrition and Women’s Bone Health

**DOI:** 10.3390/nu14040763

**Published:** 2022-02-11

**Authors:** Jose M. Moran

**Affiliations:** Metabolic Bone Diseases Research Group, Nursing and Occupational Therapy College, University of Extremadura, Avd. Universidad s/n, 10003 Caceres, Spain; jmmorang@unex.es

Nutrition is a key element that has the potential to reduce bone loss or fracture risk. This Special Issue presents some recent promising developments in nutrition and women’s bone health in the form of four original contributions and one systematic review. Overall, key aspects of the interaction between different nutrients and women’s bone health are presented through research designs of the highest quality, covering both randomized clinical trials and observational studies and headed in all cases by leading scientists in the areas of nutritional and clinical research.

By implementing a randomized, double-blind, placebo-controlled clinical trial using intention-to-treat analysis, Vallibhakara and colleagues addressed the effect of nutritional vitamin E supplementation in osteopenic postmenopausal women. This study addressed bone health by measuring changes in bone turnover markers after supplementation for 12 weeks. Most of the study participants showed good nutritional status (mean BMI 22 kg/m^2^) and high adherence was reported in both groups [1]. Those in the study group received 400 IU of mixed tocopherol on a daily basis for 12 weeks. Tablets contained 20% delta-tocopherol, 1% beta-tocopherol, 62% gamma-tocopherol and 10% alpha-tocopherol (Nat E^®^, Mega Lifesciences Public Company Limited, Samutprakarn, Thailand). While a comparison of the bone turnover markers, CTX and PINP, showed no significant difference between the vitamin E and placebo arms both at baseline and after 12 weeks of supplementation, the mean difference in the bone resorption marker, CTX, from baseline to 12 weeks was significantly different between the vitamin E group and the placebo group (−0.003 ± 0.09 and 0.121 ± 0.15, respectively (*p* < 0.001). As the bone formation markers in both groups were not significantly changed between groups in this study, Vallibhakara and colleagues suggest that the beneficial effect of vitamin E supplementation on bone health in postmenopausal women may be targeted towards slowing the increase in the bone resorption marker (CTX), which may represent the mitigation of bone loss through antiresorptive activity.

Moschonis and colleagues, following up on their previous studies on the consumption of reduced-fat Gouda cheese fortified with vitamin D3 and its efficacy in reducing the prevalence of winter vitamin D deficiency in a population of postmenopausal women in Greece [2], studied the effects of vitamin D-enriched cheese on serum PTH concentrations and selected biomarkers of bone remodeling in early or late postmenopausal women with adequate or insufficient vitamin D at baseline. Their interesting approach reveals the usefulness of supplementation of traditional dietary foods and their potential benefit for female bone metabolism [3]. A randomized, controlled, single-blinded (i.e., blinded to study participants only), controlled dietary intervention study was designed to evaluate the effect of Gouda-type cheese fortified with vitamin D3 on serum concentrations of certain calciotropic hormones (i.e., 25(OH)D, PTH), bone formation (i.e., OC, P1NP), and bone resorption markers (i.e., TRAP-5b) in postmenopausal women. The intervention proposed using vitamin D-enriched Gouda cheese significantly increased serum 25(OH)D concentrations, prevented PTH increase and reduced bone resorption in early postmenopausal women with vitamin D insufficiency. Interestingly, the reduction in bone resorption reported in women with vitamin D insufficiency coincides with increased 25(OH)D concentrations, leading the authors to propose that their results may reflect a promising nutrient-based approach to enhancing vitamin D status in these women, and to bring positive changes in bone metabolism that may be protective toward the bone loss that follows menopause.

Fatty acids are key nutrients for health, and several studies have reported an association between bone mineral density (BMD) and fatty acid intake. Robust evidence from observational studies has reported how total PUFA intake, especially n-3 and n-6 PUFA, improves BMD and even reduces the risk of fracture [4]. From an observational standpoint, Roncero-Martin and colleagues [5] evaluated the associations between serum levels of different PUFAs (n-6 and n-3), MUFAs and SFAs with bone density determined by quantitative bone ultrasound (QUS), peripheral quantitative computed tomography (pQCT) and dual-energy X-ray absorptiometry (DXA) in a sample of Spanish postmenopausal women. Independent risk factors for low BMD (T-score ≤ 1) were derived using logistic regression analysis. Higher BMI (OR = 0.893; 95% CI 0.841–0.948), *p* < 0.001) and higher plasma n-3 PUFA (OR = 0.751; 95% CI 0.587–0.960, *p* = 0.022) were identified as protective factors against low bone mass. This study reports a strong statistically independent and positive association between BMD and plasma n-3 PUFA levels, demonstrating the physiological and biochemical relevance of total plasma omega-3 fatty acids.

Based on the hypothesis that dietary total antioxidant capacity is positively associated with bone mass and negatively associated with the risk of osteoporosis in premenopausal and postmenopausal women, and based on a study of data obtained from the Korea National Health and Nutrition Examination Survey 2008–2011, Kim and colleagues [6] present data obtained from a total of 8230 female participants. The total antioxidant capacity of the diet (TAC) is a useful parameter to evaluate the overall antioxidant capacity of foods; instead of a simple addition of individual dietary antioxidants, the dietary TAC yields comprehensive information on the cumulative antioxidant capacities of different diets and may be helpful in the assessment of the preventive effects of antioxidants in different pathologies. Dietary TAC was associated positively with bone mass at the lumbar spine and femoral neck and inversely associated with the risk of osteoporosis in this cohort of postmenopausal Korean women, while in the group of premenopausal women, dietary TAC was positively associated with the bone mineral content of the lumbar spine and total femur. The results presented by Kim and colleagues in this Special Issue suggest, from an observational perspective, that consumption of high TAC foods in the diet, such as grapes, radish leaves, bell pepper paste, oranges and spinach, may improve bone health, especially in postmenopausal women, and they open the door to exciting research from the perspective of the association between the consumption of these foods and the risk of osteoporosis from an experimental point of view.

The novel advances presented in this Special Issue are further complemented by a comprehensive review of the scientific literature that offers thorough background on plant-derived compounds that potentially could be used to support bone health in perimenopausal and postmenopausal women. The review covers compounds with antiosteporotic characteristics, covering both in vitro and in vivo studies as well as clinical trials. A detailed biochemical and clinical review of the potential effects of phytoestrogens and other botanicals is provided. Słupski and colleagues [7] identify these botanicals studied as a major source of bioactive compounds, many waiting to be further investigated for their possible beneficial effects on bone health and in particular for the treatment and prevention of osteoporosis.

The current Special Issue presents progress on the topic of women’s bone health and nutrition, showing the important role that this interaction plays in human health and in different populations. The advances shown are of great interest from a clinical perspective, and these results are the starting point of what should be the basis for future research, with a growing presence of experimental studies based on different populations and ages, allowing for a deeper understanding of the ultimate interaction that takes place between nutrition and bone health in women.

## References

[1] Vallibhakara S.A.-O., Nakpalat K., Sophonsritsuk A., Tantitham C., Vallibhakara O. (2021). Effect of Vitamin E Supplement on Bone Turnover Markers in Postmenopausal Osteopenic Women: A Double-Blind, Randomized, Placebo-Controlled Trial. Nutrients.

[2] Manios Y., Moschonis G., Mavrogianni C., van den Heuvel E., Singh-Povel C.M., Kiely M., Cashman K.D. (2017). Reduced-fat Gouda-type cheese enriched with vitamin D3 effectively prevents vitamin D deficiency during winter months in postmenopausal women in Greece. Eur. J. Nutr..

[3] Moschonis G., van den Heuvel E.G., Mavrogianni C., Manios Y. (2021). Effect of Vitamin D-Enriched Gouda-Type Cheese Consumption on Biochemical Markers of Bone Metabolism in Postmenopausal Women in Greece. Nutrients.

[4] Lavado-Garcia J.M., Roncero-Martin R., Moran J.M., Pedrera-Canal M., Aliaga I., Leal-Hernández O., Martin S.R., Canal-Macias M.L. (2018). Long-chain omega-3 polyunsaturated fatty acid dietary intake is positively associated with bone mineral density in normal and osteopenic Spanish women. PLoS ONE.

[5] Roncero-Martín R., Aliaga I., Moran J., Puerto-Parejo L., Rey-Sánchez P., de la Luz Canal-Macías M., Sánchez-Fernández A., Pedrera-Zamorano J., López-Espuela F., Vera V. (2021). Plasma Fatty Acids and Quantitative Ultrasound, DXA and pQCT Derived Parameters in Postmenopausal Spanish Women. Nutrients.

[6] Kim D., Han A., Park Y. (2021). Association of Dietary Total Antioxidant Capacity with Bone Mass and Osteoporosis Risk in Korean Women: Analysis of the Korea National Health and Nutrition Examination Survey 2008–2011. Nutrients.

[7] Słupski W., Jawień P., Nowak B. (2021). Botanicals in Postmenopausal Osteoporosis. Nutrients.

